# Atypical Pediatric‐Onset Behçet's Disease Presenting With Superior Vena Cava Thrombosis in an Adolescent Male: A Case Report

**DOI:** 10.1002/ccr3.71616

**Published:** 2025-12-05

**Authors:** Ubaid Ullah, Tahreem Mari, Ubaid Ullah Mian, Aizaz Ali, Samra Aleem, Tarmim Lal, Abdul Moeez, Tanzeela Begum, Jibran Ikram

**Affiliations:** ^1^ Lady Reading Hospital Peshawar Pakistan; ^2^ Dow Medical College Karachi Pakistan; ^3^ Khyber Medical College Peshawar Pakistan; ^4^ Cleveland Clinic Foundation Cleveland Ohio USA

**Keywords:** adolescent vasculitis, Behçet's disease, dural sinus thrombosis, pathergy test, superior vena cava thrombosis, variable vessel vasculitis

## Abstract

Behçet's disease is a chronic, relapsing multisystem vasculitis known for its highly variable clinical spectrum. We report the case of an 18‐year‐old male who presented with progressive headache and facial swelling. Imaging revealed extensive thrombosis involving the dural venous sinuses, superior vena cava, and left brachiocephalic vein. Despite the absence of ocular involvement or genital ulcers, a diagnosis of Behçet's disease was established based on recurrent oral ulcers, cutaneous findings, vascular thrombosis, and a positive pathergy test, fulfilling the International Criteria for Behçet's Disease. The patient was treated with corticosteroids, azathioprine, colchicine, and rivaroxaban, resulting in clinical and radiologic improvement. This case highlights a rare and aggressive vascular manifestation of Behçet's disease in an adolescent, emphasizing the need for early diagnosis and immunosuppressive management to prevent long‐term complications.

## Key Clinical Message

Behçet's disease is a multisystem vasculitis that may lead to life‐threatening thrombosis. Clinicians should maintain a high suspicion in young patients with unexplained thrombotic events and mucocutaneous lesions. Early diagnosis and combined immunosuppression with anticoagulation are essential for optimal outcomes. Imaging is crucial for detecting vascular involvement and guiding treatment response.

## 1 Introduction

Behçet's disease (BD) is a chronic, relapsing, multisystem inflammatory disorder classified as a variable‐vessel vasculitis, capable of affecting arteries and veins of all sizes. Clinically, it presents with a wide spectrum of mucocutaneous, ocular, vascular, neurological, gastrointestinal, and musculoskeletal manifestations. First described by the Turkish dermatologist Hulusi Behçet in 1937, the disease is most prevalent along the ancient Silk Road, particularly in countries like Turkey, Iran, and Japan, but is rare in Northern Europe and the United States, indicating a strong geographic and genetic predisposition to its development [1, 2, 3]. The etiology remains poorly understood, but current hypotheses suggest a combination of genetic susceptibility most notably with HLA‐B51 and environmental triggers, possibly microbial, which activate a dysregulated immune response. Behçet's typically manifests in individuals aged 20–40 years, while pediatric onset (before 16 years) is relatively rare, accounting for only 4%–26% of cases [4].

Among the cardinal features of BD are recurrent oral aphthae, genital ulcers, skin lesions (such as acneiform eruptions and erythema nodosum), and ocular inflammation. However, the disease course can be variable, and vascular involvement, particularly venous thrombosis, is one of the most serious complications. The pathology is primarily driven by neutrophilic infiltration, endothelial dysfunction, and a hypercoagulable state, leading to thrombosis in major venous channels, including the superior vena cava, dural sinuses, and peripheral veins [5, 6]. In fact, vascular Behçet's is often underrecognized, and its manifestation can precede or mimic other disorders such as thrombophilia or systemic vasculitis. As such, early diagnosis and intervention are crucial.

Globally, the diagnosis of BD relies on clinical criteria due to the lack of specific biomarkers. The International Criteria for Behçet's Disease (ICBD) have shown higher sensitivity compared to the older International Study Group (ISG) criteria, as it does not require oral ulcers as a mandatory component [7, 8]. The clinical variability and relapsing–remitting nature of BD further necessitate individualized treatment, which may include corticosteroids, colchicine, immunosuppressants, and anticoagulation in cases with vascular involvement [9].

Data from Pakistan remain limited, with only a few case reports and small cohorts published. In a recent Pakistani cohort study, Behçet's disease was found to commonly present in young adults with a nearly equal male‐to‐female ratio, with recurrent aphthous ulcers, ocular disease, and joint pain as the most frequent symptoms [10]. However, vascular manifestations, particularly involving major veins such as the superior vena cava or dural sinuses, remain poorly documented in local literature. This case report adds to the body of evidence by describing a young male with extensive venous thrombosis secondary to BD, emphasizing the need for high clinical suspicion, early imaging, and aggressive immunosuppressive and anticoagulant therapy to prevent life‐threatening complications.

## 2 Case Presentation

### 2.1 Case History

An 18‐year‐old male presented with a 1.5‐year history of recurrent, painful oral aphthous ulcers (3–4 episodes per month), accompanied by intermittent, low‐grade fever (documented up to 38.5°C) and a progressive, holocranial headache that was throbbing in nature and worse in the mornings. He reported unintentional weight loss over this period. A notable history of pathergy was evident from the development of pustular lesions at sites of minor skin trauma, such as venipuncture. He also described the appearance of tender, purplish‐black nodular rashes on his shins, consistent with erythema nodosum. There was no history of genital ulcers, ocular pain/redness/visual changes, chronic cough, night sweats, or contact with tuberculosis.

### 2.2 Physical Examination

On examination, the patient appeared chronically ill. Vital signs were stable. He had mild periorbital puffiness and facial swelling. Multiple acneiform papulopustular lesions were noted on his face, chest, and shoulders. Dermatological examination confirmed several tender, erythematous, subcutaneous nodules on the anterior aspects of both lower limbs. The jugular venous pressure (JVP) was elevated at 8 cm, with prominent superficial venous collateral circulation visible over the anterior chest wall and neck. Cardiorespiratory and abdominal examinations were unremarkable. No active oral or genital ulcers were seen. A pathergy test, performed by intradermal saline injection, was positive at 48 h.

A formal ophthalmologic consultation was obtained to evaluate for occult ocular involvement. Slit‐lamp biomicroscopy revealed no evidence of conjunctival injection, keratic precipitates, anterior chamber cells, or flare, ruling out anterior uveitis. Dilated fundoscopy showed vitreous clarity, distinct optic disc margins without elevation or blurring, and an absence of retinal hemorrhages, exudates, or vascular sheathing. The combined findings were inconsistent with posterior uveitis, retinitis, retinal vasculitis, or papilledema.

A comprehensive evaluation for other systemic manifestations of inflammatory and vasculitic disease was undertaken. The patient denied any history of gastrointestinal symptoms such as abdominal pain, diarrhea, or hematochezia. Neurological examination revealed no focal deficits, meningismus, or signs of pyramidal involvement. Musculoskeletal review was negative for arthralgia or arthritis. There were no reported symptoms suggestive of cardiac or epididymal involvement. This targeted review of systems, in conjunction with the normal physical and neurological examination, helped to exclude subclinical involvement of the gastrointestinal, neurological, and musculoskeletal systems at the time of presentation.

### 2.3 Diagnostic Work‐Up and Management

Given the presentation of widespread venous thrombosis in a young male, a stepwise diagnostic approach was undertaken to rule out common and rare causes.

### 2.4 Initial Working Differential Diagnoses

The presentation of widespread venous thrombosis in a young male necessitated a systematic approach to investigate a broad range of differential diagnoses. Primary considerations included thrombophilia, such as inherited hypercoagulable states (e.g., Factor V Leiden or Protein C and S deficiency); malignancy, presenting as a paraneoplastic syndrome or via direct vascular compression; and chronic infections endemic to the region, notably tuberculosis and brucellosis. Furthermore, other systemic vasculitides were evaluated, including Anti‐Phospholipid Syndrome (APS) and ANCA‐associated vasculitis. Finally, given the constellation of mucocutaneous findings, Behçet's Disease (BD) was considered a leading diagnostic possibility.

Investigations were tailored to systematically evaluate the differential diagnoses. A septic workup, including blood cultures and Brucella serology, was negative. A normal procalcitonin level argued against an underlying bacterial infection. Malignancy was deemed unlikely given a normal CT abdomen and the chronic symptomatology without an identifiable mass. The absence of antinuclear antibodies (ANA) and rheumatoid factor (RF) argued against systemic lupus erythematosus and rheumatoid arthritis, respectively. While a full thrombophilia workup was not performed due to lack of availability—and anti‐phospholipid antibody and ANCA testing would have been valuable to fully exclude APS and ANCA‐associated vasculitides—the extensive, large‐vessel thrombosis in unusual sites was considered more characteristic of an inflammatory vasculitis than a simple hypercoagulable state. The results of this comprehensive diagnostic workup and Laboratory Findings are summarized in Table 1 as follows.

**TABLE 1:** Diagnostic workup and laboratory findings.

Category	Test	Result	Reference range	Interpretation/Significance
Hematological & Inflammatory	Hemoglobin	13.5 g/dL	11.5–17.5 g/dL	Normal despite chronic illness
	WBC count	7.9 × 10^3^/μL	4–11 × 10^3^/μL	Normal count with neutrophil predominance
	Neutrophils (%)	65%	40%–75%	
	Platelets	328 × 10^3^/μL	150–450 × 10^3^/μL	Normal
	ESR	45 mm/1st hr	0–15 mm/1st h	Markedly elevated, indicating active systemic inflammation
	CRP	1.8 mg/dL	< 1.0 mg/dL	Elevated, consistent with active inflammation
	Procalcitonin	0.05 ng/mL	< 0.5 ng/mL	Normal, effectively ruling out concurrent bacterial infection
Urinalysis	Pus Cells	8–10/HPF	0–5/HPF	Mild sterile pyuria, likely from genitourinary mucosal inflammation
	RBCs	Nil	0–5/HPF	No hematuria
	Protein	Nil	Negative	No proteinuria
	24‐Hour Urine Protein	126 mg/24 h	< 150 mg/24 h	Normal, excluding significant renal parenchymal involvement
	Glucose	Nil	Negative	No glycosuria
Autoimmune & Serological	ANA	Negative	Negative	Rules out Systemic Lupus Erythematosus
	Rheumatoid Factor	Negative	Negative	Rules out Rheumatoid Arthritis
	Brucella Antibodies	Negative	Negative	Excludes chronic infection like brucellosis
	Anti‐Phospholipid Abs	Not performed	—	A limitation; would have helped exclude Anti‐Phospholipid Syndrome
	ANCA	Not performed	—	A limitation; would have helped exclude ANCA‐associated vasculitides
Liver & Renal function	ALT/GPT	Normal	< 40 U/L	No hepatic involvement
	Total Protein	6.6 g/dL	6.0–8.3 g/dL	Normal synthetic function
	Serum Albumin	3.9 g/dL	3.5–5.5 g/dL	Normal
	BUN	17 mg/dL	18–45 mg/dL	Slightly low, may reflect nutritional status
	Creatinine	0.61 mg/dL	0.3–0.9 mg/dL	Normal renal function
	LDH	Not reported	140–280 U/L	Not available
Coagulation & Thyroid	PT	13.1	11–13.5 s	Normal coagulation parameter
	INR	1.09	0.9–1.2	Normal
	TSH	Normal	0.4–4.0 mIU/L	Euthyroid status confirmed

The collective investigative profile was highly indicative of an active systemic inflammatory process, consistent with Behçet's disease. This was evidenced by significantly elevated acute phase reactants (ESR 45 mm/1st h, CRP 1.8 mg/dL) in the context of a normal procalcitonin, effectively ruling out a concurrent bacterial infection. Hematologically, the patient maintained a normal hemoglobin despite chronic illness, with a mild neutrophilic leukocytosis. The urinalysis revealed a characteristic sterile pyuria, while the normal 24‐h urine protein and preserved renal function (creatinine 0.61 mg/dL) helped exclude renal parenchymal involvement typical of other vasculitides. Crucially, the autoimmune serology was negative, effectively ruling out systemic lupus erythematosus (negative ANA), rheumatoid arthritis (negative RF), and brucellosis. The preservation of liver function and euthyroid status further narrowed the differential diagnosis. While the non‐performance of a full thrombophilia panel, anti‐phospholipid antibodies, and ANCA testing remained a limitation, the overall constellation of findings marked inflammation, sterile pyuria, and a clean autoimmune profile in the presence of extensive large‐vessel thrombosis strongly supported the diagnosis of vascular Behçet's disease over alternative pathologies.

**FIGURE 1:**
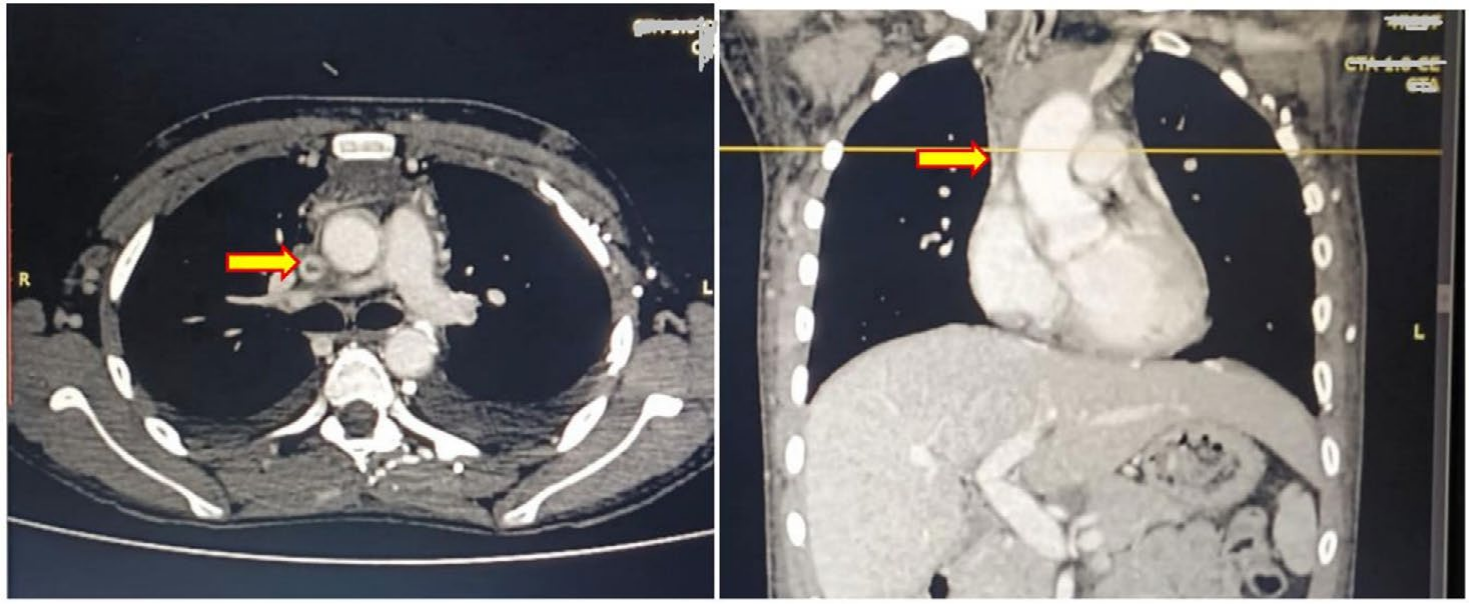
CT imaging showing a large thrombus in the superior vena cava. A large filling defect (thrombus) is visible within the lumen of the superior vena cava (SVC), as indicated by the arrow.

Imaging was pivotal. Ultrasound of the neck showed small, reactive cervical lymph nodes. CT pulmonary angiography (CTPA) confirmed extensive thrombosis of the superior vena cava (SVC) extending into the left brachiocephalic vein (Figure 1), with dilated collateral veins and no pulmonary embolism.

The constellation of recurrent oral ulcers, skin manifestations (acneiform lesions, erythema nodosum), a positive pathergy test, and extensive vascular thrombosis fulfilled the International Criteria for Behçet's Disease (ICBD), yielding a high diagnostic score.

## 3 Management

Management was initiated with high‐dose intravenous methylprednisolone (1 g daily for 3 days) to address the acute, severe vasculitis. This was followed by oral prednisolone (1 mg/kg/day) and azathioprine (2 mg/kg/day) as a steroid‐sparing agent for long‐term immunosuppression. Colchicine (0.5 mg twice daily) was added for mucocutaneous symptom control. Given the extensive thrombosis in the absence of arterial aneurysms, therapeutic anticoagulation with rivaroxaban was commenced. The patient showed significant clinical improvement within a week, with resolution of headache and facial swelling. He was discharged on a tapering dose of prednisolone, along with azathioprine, colchicine, and rivaroxaban.

At one‐month follow‐up, the patient reported resolution of all symptoms. Inflammatory markers (ESR and CRP) had normalized. A repeat CT angiography showed significant reduction in the thrombus burden within the SVC and brachiocephalic vein.

## 4 Discussion

Behçet's disease (BD) is a highly variable systemic vasculitis that displays diverse clinical manifestations across different populations, influenced by geography, genetics, and sex‐based differences. While mucocutaneous aphthosis remains its most common presentation, BD's potential for life‐threatening complications particularly major vascular involvement is underappreciated. Our case underscores one such severe and unusual manifestation: extensive venous thrombosis involving the superior vena cava (SVC), left brachiocephalic vein, and dural venous sinuses.

Vascular BD, especially venous thrombosis of large vessels, can sometimes be the initial or sole manifestation of the disease. This can mislead clinicians toward alternative diagnoses such as inherited thrombophilia, malignancy, or chronic infection [5, 11, 12]. Interestingly, unlike classical thrombotic conditions, BD‐related thrombosis arises more from vascular inflammation than from primary hypercoagulable states, a distinction highlighted by our patient's negative thrombophilia and autoimmune workups, and a normal procalcitonin level. These findings support the inflammatory origin of thrombosis in BD, in line with existing pathophysiological models involving endothelial dysfunction, neutrophilic infiltration, and overexpression of adhesion molecules [13, 14, 15, 16].

The vascular system is affected in 25%–30% of BD patients, but this involvement is the leading cause of disease‐related mortality [14]. BD uniquely targets vessels of any size and type, with a predilection for venous structures [15]. Among these, dural sinus thrombosis is rare and often underdiagnosed, typically presenting with chronic headache, papilledema, and signs of raised intracranial pressure [17, 18, 19]. Similarly, SVC thrombosis is exceedingly rare, seen in only about 2.5% of BD cases, and may result in facial swelling, pleural effusion, and dilated chest wall veins [20, 21]. The coexistence of both these vascular events in our adolescent patient is exceedingly uncommon and signifies an aggressive form of the disease.

Several reports have described large‐vessel involvement in BD, including SVC syndrome. Sarr et al. highlighted SVC thrombosis as a presenting manifestation in adolescents and young adults with BD, emphasizing that early recognition is critical [22, 23]. Zhou et al. summarized cases of vena cava involvement in BD and noted that patients often present with extensive thrombotic disease requiring prolonged immunosuppression, with or without anticoagulation [21]. Compared with these reports, our patient was unusual in demonstrating both SVC and dural sinus thrombosis concurrently, which is rarely documented in pediatric‐onset BD.

Cerebral venous sinus thrombosis (CVST) is among the most frequent neuro‐Behçet manifestations, with an estimated prevalence of 10%–20% in BD cohorts [15]. Shi et al. reported that dural sinus involvement is often chronic, with extensive collateralization, similar to the MRV findings in our case [15]. While most reported patients are in their third or fourth decade, our patient developed CVST in adolescence, underscoring the aggressive vascular course of pediatric‐onset BD.

Adding to the diagnostic challenge, our patient lacked two of the classical hallmarks of BD genital ulcers and ocular involvement despite fulfilling the International Criteria for Behçet's Disease (ICBD). This case thus highlights the limitations of traditional diagnostic expectations and the strength of ICBD, which allows for diagnosis based on weighted scoring across various systemic features. In our case, the patient met the diagnostic threshold through a combination of mucocutaneous findings, neurological involvement, vascular thrombosis, and a positive pathergy test [22, 23].

What makes this presentation particularly noteworthy is the age of the patient. BD typically presents between ages 20 and 40, with pediatric‐onset cases (before age 16) accounting for just 4%–26% of all patients [5, 6]. Our case involved an 18‐year‐old male on the cusp of pediatric‐onset BD, emphasizing the need to consider BD in younger patients presenting with unexplained systemic and vascular symptoms. As reported by Khan et al. in a retrospective study from Pakistan, BD in local populations tends to manifest in middle‐aged adults, with vascular complications being infrequent [7]. Our case not only deviates from that pattern but also highlights the broader spectrum of disease expression in South Asian patients.

The mucocutaneous findings in our case—recurrent oral ulcers, erythema nodosum‐like lesions, and acneiform eruptions are consistent with BD. However, the absence of genital ulcers and uveitis posed a diagnostic hurdle, demonstrating the importance of maintaining a high index of suspicion in cases where classical features are incomplete [1, 8, 9]. According to a large Chinese study, oral aphthous ulcers remain the most frequent clinical sign, followed by genital ulcers and erythema nodosum, with genital ulcers occurring more frequently in younger patients [9, 10]. Our case highlights how atypical presentations may still represent BD and should prompt thorough investigation when other features align.

The pathergy test, which was positive in our patient, continues to play a valuable role in diagnosis, especially in endemic regions. Although its sensitivity has declined over time, it remains an important supportive feature in the ICBD algorithm [22, 23, 24]. High‐resolution imaging, including magnetic resonance venography (MRV) and CT pulmonary angiography (CTPA), proved indispensable in this case. These modalities not only confirmed the presence of thrombosis but also revealed well‐developed collateral circulation, suggesting chronicity and helping explain the absence of overt neurological deficits despite extensive dural sinus occlusion [12].

Therapeutically, there remains controversy over the use of anticoagulation in vascular BD because of the risk of pulmonary arterial aneurysm rupture. Nonetheless, multiple reports suggest that in the absence of pulmonary arterial involvement, a combination of high‐dose corticosteroids, immunosuppressive therapy, and cautious anticoagulation may improve outcomes [16, 19]. Immunosuppressive therapy remains the cornerstone of treatment. In our patient, pulse corticosteroids were initiated acutely, followed by azathioprine and colchicine for long‐term immunomodulation. Colchicine remains the first‐line therapy for mucocutaneous involvement, while azathioprine has proven efficacy in preventing relapses in systemic disease [24]. Although anticoagulation remains controversial in vascular BD, our patient had no aneurysmal disease, permitting the use of rivaroxaban, which contributed to favorable outcomes [24].

This case illustrates how early and aggressive intervention including immunosuppressants and anticoagulants can lead to marked clinical and radiologic improvement. The absence of neurological deterioration despite extensive cerebral venous involvement further underscores the protective role of early collateral formation and therapeutic intervention. By documenting concurrent SVC and dural sinus thrombosis in adolescent‐onset BD, this report adds to the limited literature and highlights the need for heightened vigilance, structured diagnostic work‐up, and prompt immunosuppressive treatment in atypical or incomplete cases of BD.

A few case reports and small series have documented SVC syndrome or SVC thrombosis in Behçet's disease; Sarr et al. [20] described SVC involvement as a presenting manifestation, and Zhou et al. [21] summarized risk factors and outcomes for vena cava syndrome in BD. Likewise, cerebral venous sinus thrombosis has been reported in BD cohorts and case series (e.g., Shi et al. [18]). Compared with these reports, our patient is notable for the coexistence of chronic dural sinus thrombosis and extensive SVC/brachiocephalic thrombosis at a younger age. Management in the reported cases varied, but favorable outcomes were achieved with early immunosuppression and careful use of anticoagulation when no pulmonary arterial aneurysm was present—an approach mirrored in our case. To further contextualize these findings, we reviewed previously published cases of SVC and dural sinus thrombosis in BD, and the key demographic features, vascular sites involved, imaging modalities, treatment strategies, and outcomes are summarized in Table 2, highlighting the similarities and differences between our patient and reported cases.

**TABLE 2:** Comparison of present case with published cases of superior vena cava and dural sinus thrombosis in Behçet's disease.

Author/Year	Age/Sex	Vascular site(s) involved	Imaging modality	Treatment (main)	Outcome
Sarr et al. (2015)	21 y, M	Superior vena cava thrombosis	CT venography	Corticosteroids + azathioprine	Symptomatic improvement, venous recanalization
Zhou et al. (2022)	24 y, M	Vena cava syndrome (SVC + IVC)	CT/MR venography	Cyclophosphamide + steroids ± anticoagulation	Partial recanalization, clinical remission
Shi et al. (2018)	29 y, F	Cerebral venous sinus thrombosis (transverse & sigmoid)	MRV	Corticosteroids + colchicine	Improved headache, recanalization on follow‐up
Other BD series	18–40 y, mixed	Cerebral venous sinus thrombosis	MRV	Corticosteroids ± azathioprine, anticoagulation in selected	Variable, most improved with immunosuppression
Our case (2025)	18 y, M	SVC + left brachiocephalic vein	CT chest	IV methylprednisolone pulse → oral prednisolone + azathioprine + colchicine; rivaroxaban (after excluding aneurysm)	Resolution of facial swelling, stable neuro status, radiologic improvement

## 5 Conclusion

Dural sinus and SVC thrombosis, though rare, seem to be known complications of BD. In the absence of genital ulcers or ocular symptoms, this case illustrates an uncommon pediatric presentation of Behçet's illness that includes both dural sinus and SVC thrombosis. This highlights the diversity of BD and stresses how crucial it is to keep a high level of suspicion when dealing with unusual presentations to initiate prompt treatment. The course of the disease can be significantly changed by early identification and suitable immunosuppressive treatment, which lowers the chance of long‐term consequences. As juvenile BD with significant vascular consequences remains underreported, this case provides valuable insights into the clinical spectrum of BD and highlights the need for tailored treatment in enhancing prognosis.

## Author Contributions

**Ubaid Ullah:** visualization, writing – original draft, writing – review and editing. **Tahreem Mari:** writing – original draft, writing – review and editing. **Ubaid Ullah Mian:** writing – original draft, writing – review and editing. **Aizaz Ali:** writing – original draft, writing – review and editing. **Samra Aleem:** writing – original draft, writing – review and editing. **Tarmim Lal:** investigation, supervision, visualization. **Abdul Moeez:** supervision, visualization, writing – original draft. **Tanzeela Begum:** supervision, visualization, writing – review and editing. **Jibran Ikram:** validation, writing – original draft, writing – review and editing.

## Funding

The authors have nothing to report.

## Ethics Statement

Ethics approval was taken from the relevant institution for the publication of this case report.

## Consent

Written informed consent was obtained from the patient for publication of this case report and any accompanying images. The Wiley standard patient consent form was used.

## Conflicts of Interest

The authors declare no conflicts of interest.

## Data Availability

The data supporting the findings of this case report are available from the corresponding author upon reasonable request.
